# Visual search behavior and performance in luggage screening: effects of time pressure, automation aid, and target expectancy

**DOI:** 10.1186/s41235-021-00280-7

**Published:** 2021-02-25

**Authors:** Tobias Rieger, Lydia Heilmann, Dietrich Manzey

**Affiliations:** Department of Psychology and Ergonomics, Chair of Work, Engineering, and Organizational Psychology, F7, Technische Universität Berlin, Marchstr. 12, 10587 Berlin, Germany

**Keywords:** Time pressure, Visual search, Automation support, Target expectancy, Luggage screening

## Abstract

Visual inspection of luggage using X-ray technology at airports is a time-sensitive task that is often supported by automated systems to increase performance and reduce workload. The present study evaluated how time pressure and automation support influence visual search behavior and performance in a simulated luggage screening task. Moreover, we also investigated how target expectancy (i.e., targets appearing in a target-often location or not) influenced performance and visual search behavior. We used a paradigm where participants used the mouse to uncover a portion of the screen which allowed us to track how much of the stimulus participants uncovered prior to their decision. Participants were randomly assigned to either a high (5-s time per trial) or a low (10-s time per trial) time-pressure condition. In half of the trials, participants were supported by an automated diagnostic aid (85% reliability) in deciding whether a threat item was present. Moreover, within each half, in target-present trials, targets appeared in a predictable location (i.e., 70% of targets appeared in the same quadrant of the image) to investigate effects of target expectancy. The results revealed better detection performance with low time pressure and faster response times with high time pressure. There was an overall negative effect of automation support because the automation was only moderately reliable. Participants also uncovered a smaller amount of the stimulus under high time pressure in target-absent trials. Target expectancy of target location improved accuracy, speed, and the amount of uncovered space needed for the search.

*Significance Statement* Luggage screening is a safety–critical real-world visual search task which often has to be done under time pressure. The present research found that time pressure compromises performance and increases the risk to miss critical items even with automation support. Moreover, even highly reliable automated support may not improve performance if it does not exceed the manual capabilities of the human screener. Lastly, the present research also showed that heuristic search strategies (e.g., areas where targets appear more often) seem to guide attention also in luggage screening.

Visual search plays an important role in a plethora of safety-critical real-world tasks. One important example involves luggage screening at airport security checkpoints in order to ensure the safety of thousands of travelers worldwide every day (Biggs et al. 2018; Biggs and Mitroff 2014; Schwaninger et al. 2005). For this purpose, luggage screeners have to check X-ray images of baggage items for critical targets such as knives, weapons, or explosive materials. In order to do this, they search for these targets (e.g., a knife) among visual background noise, consisting of objects from everyday life (e.g., a pen). That is, they essentially perform a visual search task as has been frequently studied in fundamental cognitive research. However, in evaluating the visual search performance of luggage screeners, several specific characteristics of this workplace should be taken into account and one such factor is time pressure.

Time pressure is a ubiquitous factor in a lot of work settings, and this is often true for luggage screeners as well. For instance, there might be different airports where time pressure on operators varies due to differences in volume of traffic. Even within one airport, there are busier and less busy times, e.g., related to time of day or other situational circumstances (such as a global pandemic) affecting the work rate at which screeners have to work from time to time. In the human factors literature and outside of highly controlled laboratory environments, time pressure is usually considered as a to-be-avoided workload factor (e.g., Carayon and Gurses 2008; Hendy et al. 1997; Moray et al. 1991) because it can impair performance and increase workload. Regarding general performance consequences in controlled laboratory experiments, there is usually a speed–accuracy tradeoff in visual search tasks (e.g., Drury 1994; Wickelgren 1977) which has also been shown in a luggage screening task (e.g., McCarley 2009).

Some straightforward theoretical predictions about the effects of time pressure in a visual search task such as luggage screening can be derived from the self-terminating (SST) model of visual search (e.g., Bricolo et al. 2002; Pashler 1987; Snodgrass 1972; Treisman and Gelade 1980; van Zandt and Townsend 1993). This model assumes that visual search proceeds serially (i.e., one search item at a time) and is terminated once an item is found to be a target item (i.e., self-termination). Consequently, target-present decisions are usually faster than target-absent decisions (e.g., Treisman and Gelade 1980; Wolfe 1998). With respect to the specific effects of time pressure on target-present versus target-absent decisions, this model would predict an interaction effect of target presence with time pressure for response times. That is, because target-absent responses are much slower than target-present responses, restricting the time available should mainly shorten those target-absent response times (i.e., an interaction between time pressure and target presence reflected in a stronger increase in response speed for target-absent than for target-present trials under high time pressure).

A second factor which might affect visual search performance in luggage screening is automation. In recent years, automated decision support systems (DSSs) have been introduced to support screeners detect potential threat items (e.g., Chavaillaz et al. 2018, 2019; Hättenschwiler et al. 2018; Huegli et al. 2020). Based on an automated image analysis, the DSS provides the operator with an automated recommendation about the true state of the world (in the current case, information whether a bag contains a target or not). However, the final decision is usually left with the operators. Generally, the goals of introducing an automated DSS into any context are improving overall performance as well as safety and reducing the operator’s workload (Chavaillaz et al. 2018; Hättenschwiler et al. 2018; Mosier and Manzey 2020). One specific prediction concerning the effect of automation support on luggage-screening performance again can be derived from the SST model. This model would predict that the difference in response time between target-present and absent responses decreases with the automation available compared to manual performance. That is, for target-absent trials, one would assume that participants terminate their search earlier if they have an automated decision aid’s recommendation available to help inform their decision. Conversely, for target-present trials, when just using a general cue informing operators about the presence of a target without precisely locating it (like we do in the present study), one would not expect the participants to find the target much faster than when working manually, i.e., without automation support.

Unfortunately, however, DSSs are not always used appropriately, potentially leading to automation disuse and misuse (Parasuraman and Riley 1997), and an overall performance which is less-than-ideal (Bartlett and McCarley 2017; Meyer 2001). In addition, effects of automation support on performance in visual search tasks seem to interact with effects of time pressure. More specifically, being under extreme time pressure has been shown to increase operator reliance on and compliance with the automation (Rice et al. 2008; Rice and Keller 2009; Rice et al. 2010; Rice and Trafimow 2012). Thus, somewhat counterintuitively, time pressure might not necessarily be detrimental in this case but could even increase the overall visual search performance (i.e., accuracy and sensitivity) if humans more strictly follow the DSS’s recommendations under high time pressure—and if the automation alone were more accurate and sensitive than the human.

Finally, another potentially influencing factor that might have an effect on visual search performance in luggage screening is probability cueing which also has been extensively studied in basic cognitive research. It is conceivable that screeners have had the experience of threat items being hidden in potentially concealing areas of bags more often than in other areas and screeners could take on the search strategy to check these areas first. In spatial probability cueing paradigms, targets show up more frequently in one specific region of the search area compared to other search regions. For example, in the study of Jiang et al. (2013), participants searched for a target letter among distractor letters. During training, the target letter appeared more often in one of four search quadrants. After training, the target letter appeared in all four quadrants completely random. However, participants detected the target faster if it appeared in the same quadrant as in the previous training trials. Several other studies (Chun and Jiang 1998; Geng and Behrmann 2005; Hoffmann and Kunde 1999; Miller 1988; Umemoto et al. 2010) have shown similar findings, suggesting that a high probability of a target appearing in congruent location can lead to faster and more accurate responses, as attention is selectively focused on this respective location like a spotlight (Miller 1988). Several studies suggest that such target expectancy effects are not limited to basic laboratory tasks but can also be found in more complex visual search tasks in the real world. For instance, visual search tasks in a medical context (such as mammography) have investigated expectancy effects (e.g., Sha et al. 2018) and extended the basic findings mentioned above to this applied area. Moreover, research focusing on expertise in radiological images (Kundel and Toto 1972; Nodine et al. 1996) has found that experts tend to concentrate more clearly on the abnormal features—something that likely is also connected to target expectancy. Given that luggage screening and medical image perception share many commonalities (Gale et al. 2000), it seems likely that this kind of target expectancy effect can be replicated in the current context of luggage screening. Moreover, effects of target expectancy have also been found in driving contexts (Pollatsek et al. 2006; Shinoda et al. 2001) and other more applied contexts (e.g., Brockmole and Henderson 2006; Mack and Eckstein 2011; Oliva et al. 2004).

The present study aims at investigating the impact of these potential performance-shaping factors mentioned above (i.e., time pressure, automation support, and target expectancy) and, more importantly, their possible interactions on the performance on a typical luggage-screening task. We are interested to what extent the results of earlier laboratory research and predictions of the SST apply also for screening of complex luggage images. Moreover, to the best of our knowledge, target expectancy effects have not yet been directly demonstrated in the context of luggage screening. Further, even though a replication of known basic effects of visual search with luggage screening tasks might not be a big surprise, we are particularly interested in possible interaction effects between these different factors, as they can provide important insights to better assess the possible performance consequences (and potential safety risks) arising from different context conditions in luggage screening.

## The present experiment

To investigate the impact of time pressure, automation support, and target expectancy in the context of luggage screening, we used a visual search paradigm where participants used the mouse to sequentially uncover small portions of the stimuli which consisted of a set of X-ray images typical for luggage screening (for full description see Procedure). This allowed us not only to measure accuracy and response times, but also to check how extensively the participants searched through the luggage image prior to making their decision. Note that the predictions from the SST model mentioned above should also hold true for the search amount uncovered which should provide a more direct measure about the scope of the visual search. However, to allow participants guidance as would be the case in a normal visual search task (e.g., Wolfe 2007), we briefly displayed the full stimulus before the mouse-search started.

Our mouse-over approach corresponds in some way to the paradigms used by Drew and Williams (2017) and Peltier and Becker (2017) who also assessed visual search performance by uncovering techniques. Using eye tracking, both studies assessed which areas of complex stimuli (i.e., outdoor scenes and 1/f noise in Drew and Williams (2017), ‘Where’s Waldo?’ images in Peltier and Becker (2017)), had been previously fixated and reported this information live back to the participants. The goal of these studies was to check whether providing this kind of live feedback to participants was helpful for discovering targets, both these studies concluded that this kind of eye-movement feedback was not helpful. Even though the dynamic uncovering of a complex image represents a commonality with our approach, the present study has several differences to those studies, as a) we did not use eye tracking and b) the total area uncovered was used as a dependent variable and was not fed back to the participants during the search.

With respect to the impact of time pressure and automation support on accuracy, response time (RT), and search amount, we hypothesized that time pressure decreased all of these performance variables due to a time-pressure-induced speed-accuracy tradeoff. However, whether a reliable (85%) automation is available or not might make a difference here, with automation support potentially decreasing the negative effects of time pressure or even causing positive effects of time pressure (e.g., Rice and Keller 2009). With respect to target expectancy effects, we expected that they can also be extended to luggage screening. Here, we hypothesized that responses would be more accurate, faster, and needed a smaller search amount when targets appeared in a predictable location than when they did not. As was argued above, time pressure might induce more heuristic search strategies and might therefore potentially also increase such target expectancy effects. We had no clear hypothesis regarding what role the presence of an automated DSS might play for potential target expectancy effects, particularly because the DSS’s advices only cued whole images instead of specific locations.

## Method

### Participants

Forty-eight novice participants took part in the experiment, but three participants in the high-time-pressure condition had to be excluded from further data analyses because they either did not respond at all or did not uncover any part of the screen prior to their decision in the second half of the experiment. Thus, the final sample consisted of 45 participants (26 females), aged between 22 and 40 years (*M* = 27.7). Participants signed written informed consent and were compensated for their study participation via course credit or nine euros. The study was approved by the local ethics committee.

### Apparatus and Stimuli

Stimuli were grayscale luggage X-ray images taken from the X-ray ORT 1.3 and X-ray ORT 2.0 (Hardmeier et al. 2005; Schwaninger et al. 2005). All images were resized to 714 × 562 pixels. We used 32 unique target-present images. Target-present stimuli always included one target (gun or knife), with a low level of superposition, an easy viewpoint, and a high level of bag complexity (see Hardmeier et al. 2005; Schwaninger et al. 2005). The quadrant in which the target was located was determined for each target-present image. Only if the quadrant could not be determined clearly, the image was edited using GNU Image Manipulation Program (GIMP) to digitally insert a target to a suitable location. During this editing process, we took great care in not changing brightness, contrast, transparency, shading, or possible overlap. Target-absent images were the same stimuli but without the target present. To increase the overall number of stimuli and to prevent recognition effects, each image was not only used in its original orientation but also rotated by 90°, 180°, and 270°, resulting in four different versions of each image. The final image set, thus, consisted of 256 luggage X-ray images: 32 unique-target-present images * 2 (corresponding target-absent image) * 4 (image rotation).

We used a custom-built Java program to run the experiment. Workstations for participants consisted of standard Windows computers, with a 24-inch screen and a 1920 × 1200 resolution. Responses were made using the ‘q’ (target-absent) and ‘w’ (target-present) keys.

### Procedure

Participants were randomly assigned to either the low or high time-pressure condition in equal numbers. The experiment consisted of an initial practice phase without the mouse-over search followed by the main experimental phase with the mouse-over search.

In order to familiarize the participants with the target items and stimulus material, they were first shown target overview images (i.e., two gun and two knife overview images) for 10 s each. Then, they were shown 16 images from the ORT 1.3 and 2.0 (eight target-present and eight target-absent images in random order) and had to decide whether or not a target was present. Each of these practice trials started with a fixation cross for 1000 ms, followed by the presentation of the stimulus for a maximum of 8 s or until a response was made. Directly upon their response, participants received on-screen feedback about the correctness of their response for 1000 ms, followed by a 1000 ms blank screen inter-trial interval. The following practice block consisted of 60 other images from the ORT 1.3 and 2.0 and was not used in the main part of the experiment. Only cumulative feedback (% correct) after completion was provided for these 60 trials. During the whole practice phase, participants always saw the entire images, i.e., were not required to use the mouse-over search procedure. This was done in order to allow participants to get to know what luggage X-ray stimuli generally look like in full view.

The following main experimental phase was introduced by again providing the images of the specific targets which were contained in the target-present images. The data collection was then split into two halves, differing in whether or not automation support was provided for the task. The order of these halves was counterbalanced across participants.

Each half started off with familiarization trials to allow participants to get comfortable with the mouse-over search functionality, and the automation support, respectively. The first half (either with or without automation support) always included a total of 12 familiarization trials, with a first set of six trials presented for 30 s and a second set of six trials presented according to the time restriction of that participant’s time-pressure condition. The second half always included only the latter set of six trials with the corresponding time restriction for each subject. Whether or not an automation aid was given in these trials was always matched to the subsequent experimental blocks.

Following these familiarization trials, two blocks of 60 trials each were presented in each half, resulting in a total of 240 trials. In every block, half the trials were target-present trials and half the trials were target-absent trials. Out of the target-present trials, 70% included stimuli where the target was presented in the same quadrant of the image (target-often location). The remaining 30% of target-present trials included stimuli where the target location was equally distributed across the other three quadrants of the image. The target-often locations used in target-present trials differed between the two halves of the experiment and were also counterbalanced across participants.

The same luggage pieces were presented no more than two times per block but were never presented in the same orientation within one block (i.e., even with two identical bags, in different-orientation images the target would be in a different quadrant). Overall, there were eight different trial lists with two different list versions for each target-often location. Repetitions were controlled for as target-often locations and corresponding trials lists were counterbalanced between participants and time-pressure condition, in their order of presentation and in their coupling order with the automation condition. Additionally, per experimental half, automation mistakes never occurred at the same unique image (i.e., regardless of orientation).

**Fig. 1:**
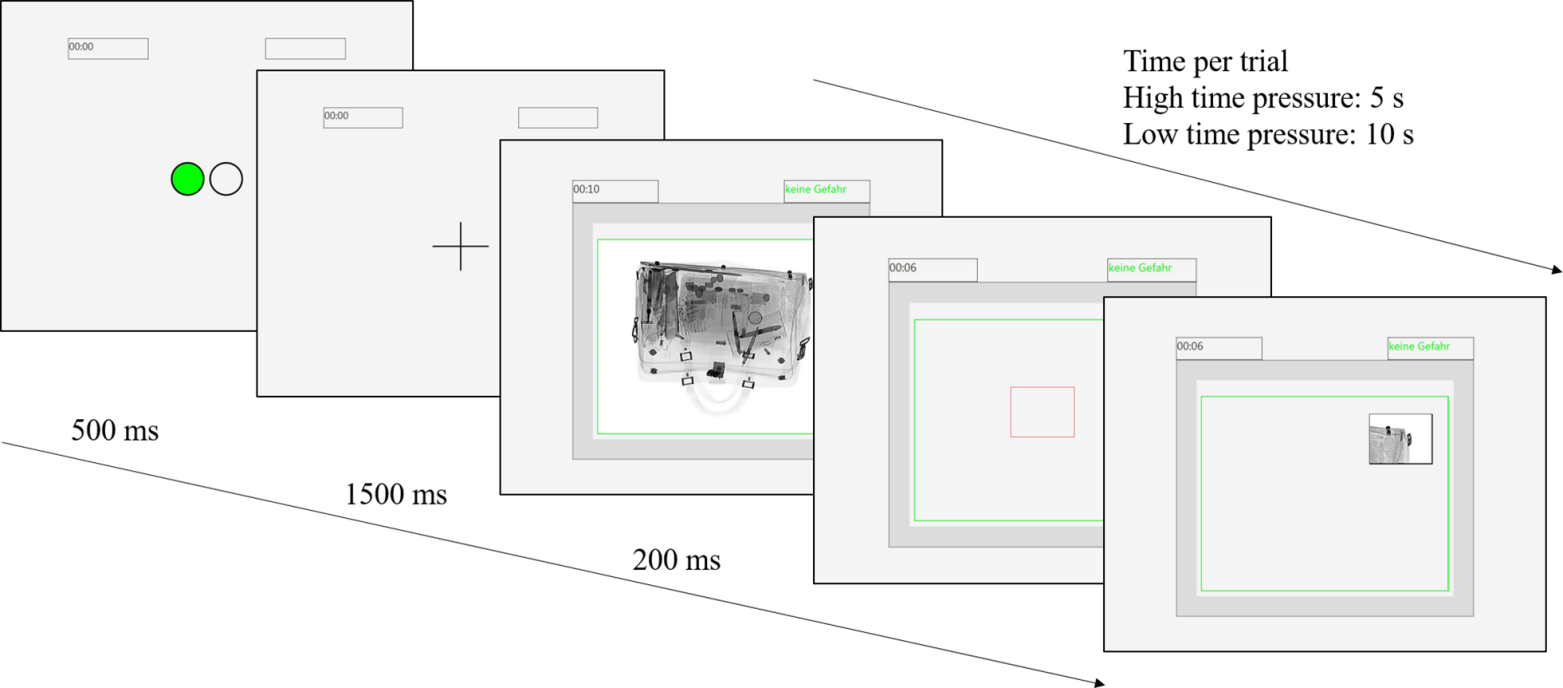
Typical trial procedure in the automation condition. Note that in the manual blocks, the fixation cross was presented for 2000 ms with no automation cue at trial onset

During all trials of the main experiment, participants were required to use the mouse-over procedure to inspect the stimuli. The structure of each trial is visualized in Fig. 1. In the half with automation support, each trial started off by indicating the DSS’s recommendation, indicated by either a green filled circle (target absent) or a red filled circle (target present). Compatible with the locations of the response keys (‘q’ for target absent and ‘w’ for target present) on the keyboard, green circles were shown on the left and red circles on the right. This recommendation was present for 500 ms, after which a fixation cross was shown for 1500 ms. In the manual blocks, the fixation cross was presented for 2000 ms to ensure equal task preparation times in both conditions. Afterwards, the stimulus was shortly displayed in full view for 200 ms and the countdown for this trial started in the upper left corner above the stimulus (5 s in the high-time-pressure condition, 10 s in the low-time-pressure condition). In the automation condition, a rectangle colored in the color of the advice (either green or red) surrounded the stimulus display area and the cue *Keine Gefahr* (no threat) or *Gefährlicher Gegenstand* (threat item) was displayed on the upper right side of the stimulus display. After the first 200 ms, the area where the stimulus was presented was grayed out, and a red-lined rectangle measuring 180 × 142 pixels appeared in the middle of the image display area. The size of the search area was chosen at a size where it was theoretically possible to view the targets in full within the search area. The rectangle could be moved freely across the image area by mouse-cursor to start the search at the preferred location. Upon mouse-click, the rectangle’s border color changed to black and an X-ray image section appeared behind the rectangle. Upon movement, previously uncovered areas were grayed out again. As soon as a key was pressed, the next trial started. If there was no response after the countdown had elapsed, the next trial started. Participants were instructed that not responding in time was counted as a target-absent response. Participants received cumulative feedback (% correct responses) after every block.

In the half with the automated DSS, there were eight false alarms and one miss per block, resulting in an overall automation accuracy of 85%. Making the DSS false-alarm prone was deliberately chosen because real-world automations in safety-critical contexts usually follow a safe-engineering approach, trying to avoid misses of crucial events. Automated support systems currently used at airports have a similar accuracy (Hättenschwiler et al. 2018; Mery et al. 2013). False alarms only occurred after some trials, so participants could build trust in the automated diagnostic aid (Lee and See 2004). Misses only appeared toward the end of the block. Specifically, false alarms did not occur in the first ten trials of each block and were at least three trials apart from each other. The respective misses occurred in trials 56 and 58, with one miss per automation block. Participants were told that the automated diagnostic aid might miss a threat or indicate one even when none was actually present. They were advised to decide on their own to follow the automated recommendation or not.

After each experimental half, participants filled out the NASA Task Load Index (NASA-TLX; Hart and Staveland 1988). Moreover, after the experimental half with the automation, participants also filled out the ‘Fragebogen zur mehrdimensionalen Erfassung von Vertrauen’ (FMV; Wiczorek 2011). This questionnaire measures overall trust and trust on the dimensions perceived reliability, utility, intention, and transparency on a four-point Likert scale. Because the focus of the present manuscript is on visual search, the data from these questionnaires are given in “Appendix.” Overall, the experiment lasted about 60 min per participant.

### Design

Time pressure was varied between subjects (i.e., high vs. low). The factor automation support (i.e., automation vs. manual) and the factor target presence (i.e., present vs. absent) were varied within subjects. For the target-present trials, there was an additional within-subjects factor, that is, target-location congruency (i.e., congruent vs. incongruent).

## Results

### Time pressure and automation support

#### Performance

**Fig. 2:**
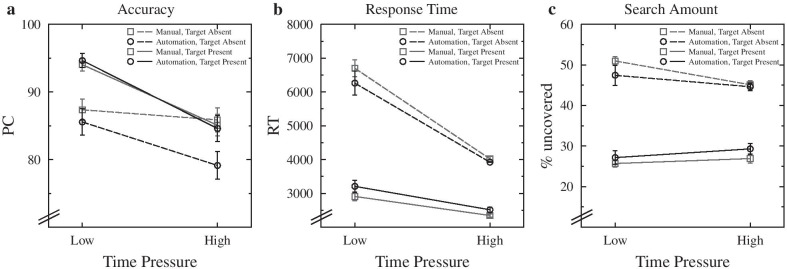
Data for percent correct (**a**), response times (**b**), and search amount (**c**), separately for each time-pressure condition, as a function of automation condition and target presence. Error bars represent standard errors of the mean. PC: percent correct, RT: response time

Accuracy was assessed by the percentage of correct responses (PC) as the overall performance measure. The results for PC are visualized in Fig. 2a. There was a significant main effect of time pressure, *F*(1,43) = 24.751, *p* < 0.001, *η*_*G*_^*2*^ = 0.166, with more accurate responses under low (90.4%) than under high (83.7%) time pressure. Descriptively, this effect seemed to be mainly linked to a time-pressure-induced increase of misses (PC difference low vs. high time pressure for target present: 9.4%) and only to a lesser degree to an increase in false alarms (difference for target absent: 4.0%). The main effect of automation condition was also significant, *F*(1,43) = 6.894, *p* = 0.012, *η*_*G*_^*2*^ = 0.019, with more accurate responses in the manual (88.3%) than in the automation (86.3%) condition. Moreover, there was also a significant main effect of target presence, *F*(1,43) = 12.249, *p* = 0.001, *η*_*G*_^*2*^ = 0.103, with more accurate responses for target-present (89.9%) than for target-absent (84.6%) trials. The interaction of target presence and automation support was also significant, *F*(1,43) = 6.773, *p* = 0.013, *η*_*G*_^*2*^ = 0.020, with a much larger difference between target-present and absent PC for the automation condition (7.4%) than for the manual condition (3.2%). No other effect in the ANOVA was significant, *p*s > 0.064.

To complete the performance picture, we also calculated the corresponding performance in terms of signal detection theory measures, i.e., sensitivity (d_a_, as specified in Sterchi et al. 2019) and the response criterion C (for calculation see Stanislaw and Todorov 1999). As some condition x participant combinations produced perfect hit rates, we loglinear corrected all hit and false alarm rates (Hautus, 1995). Note that for the DSS alone, d_a_ was 2.59 and C was −0.564. We ran an ANOVA for both measures with time pressure as the between-subject factor and automation support as the within-subject factor.

For sensitivity, there was a significant main effect of time pressure *F*(1,43) = 45.103, *p* < 0.001, *η*_*G*_^*2*^ = . 406 with worse sensitivity under high (1.96) than under low (2.81) time pressure. Neither the main effect of automation support (*p* = 0.534) nor the interaction (*p* = 0.183) was significant.

For the response criterion, there was a significant main effect of time pressure, *F*(1,43) = 7.224, *p* = 0.010, *η*_*G*_^*2*^ = 0.114, with a shift to a more conservative response criterion under high (-0.048) than under low (-0.244) time pressure. The main effect of automation condition was also significant, *F*(1,43) = 6.675, *p* = 0.013, *η*_*G*_^*2*^ = 0.035, with a more liberal response criterion for the overall human–automation system with the automation available (-0.204) than in the manual condition (-0.101). The interaction was not significant (*p* = 0.580).

#### Response times

Response time (RT) was measured as the time from initial X-ray image onset (i.e., onset of the 200-ms preview) to key press. One participant from the high-time-pressure condition was excluded for this analysis because she/he did not produce any RTs for target-absent trials but always just waited until the time had elapsed. We only used RTs from correct trials and only trials where a response was given. After visual inspection of the distribution of the data, we excluded all trials with responses faster than 750 ms and slower than 9250 ms. After applying all these exclusion criteria, 73.7% of total trials were included for the analysis. The means of the final sample for the different conditions are shown in Fig. 2b.

The main effect of time pressure was significant, *F*(1,42) = 56.746, *p* < 0.001, *η*_*G*_^*2*^ = 0.433, with faster RTs in the high time pressure (3201 ms) than in the low time pressure (4769 ms) condition. Moreover, the main effect of target presence was also significant, *F*(1,42) = 426.789, *p* < 0.001, *η*_*G*_^*2*^ = 0.657, with considerably faster responses when a target was present (2773 ms) than when no target was present (5339 ms). The interaction of time pressure and target presence was also significant, *F*(1,42) = 61.344, *p* < 0.001, *η*_*G*_^*2*^ = 0.216, indicating a larger difference between target-present and absent trials under low-time-pressure (difference: 3421 ms) than under high-time-pressure (difference: 1541 ms). Finally, also the interaction of automation support and target presence was significant, *F*(1,42) = 17.258, *p* < 0.001, *η*_*G*_^*2*^ = 0.019, with a larger difference between target-present and target-absent trials in the manual (2829 ms) than in the automation (2304 ms) condition. All other effects in the ANOVA were not significant, *p*s > 0.059.

#### Search amount and search speed

Our paradigm also allowed us to track exactly how much of the screen participants uncovered during their search, and we also analyzed these data with an ANOVA parallel to the RT analysis, but without excluding any trials. By analyzing the mouse tracking data, we were able to recover the percentage of uncovered screen for each trial, i.e., *Search Amount*. Note that the absolute values of search amount are not particularly informative here because some parts of the images were not really worth searching in because the bags did not fully fill out the image display; instead, the differences between the conditions are particularly interesting and one should therefore focus on those. The corresponding means of the different conditions are shown in Fig. 2c.

The ANOVA revealed a significant main effect of target presence, *F*(1,43) = 805.472, *p* < 0.001, *η*_*G*_^*2*^ = 0.685, with a much larger search amount for target-absent (47.2%) than for target-present (27.2%) trials. Moreover, the interaction of time pressure and target presence was also significant, *F*(1,43) = 18.634, *p* < 0.001, *η*_*G*_^*2*^ = 0.048, with a larger difference between target-present and absent trials in the low-time-pressure condition (22.7%) than in the high-time-pressure condition (16.8%), indicating a less exhaustive search with earlier termination under time pressure particularly in trials with a target being absent. Finally, also the interaction of target presence and automation support was significant, *F*(1,43) = 18.428, *p* < 0.001, *η*_*G*_^*2*^ = 0.021, with a larger difference in search amount between target-present and absent trials in the manual condition (22.0%) than in the automation condition (18.0%). As becomes evident from Fig. 2c, participants terminated their search in target-absent trials earlier and with less space uncovered when they had an automation available than when they performed the task manually. No other effect was significant, *p*s > 0.257.

As an additional exploratory analysis, we also analyzed *Search Speed*, defined as the percentage of screen uncovered per second (from the start of the search) in order to directly evaluate the combined effects of time pressure and automation support on search amount and response speed. In these analyses, we only included trials where an actual search was done, i.e., excluding trials with a search amount of zero. Other than that, the search speed analyses were parallel to the search amount. This also meant we had to drop an additional participant in this search speed analysis, as one participant never searched for a target with the automation available in target-absent trials.

The ANOVA revealed a significant main effect of time pressure, *F*(1,42) = 23.325, *p* < 0.001, *η*_*G*_^*2*^ = 0.277, that is, under high time pressure, participants uncovered space faster (14.69%/s) than under low (11.34%/s) time pressure. There also was a significant main effect of target presence, *F*(1,42) = 89.592, *p* < 0.001, *η*_*G*_^*2*^ = 0.225, with a higher search speed (14.43%/s) for target presence than for target-absent trials (11.46%/s), possibly indicating that the search speed slowed down over the trial when there was no target found in a serial search of the image. The interaction of time pressure and target presence was also significant, *F*(1,42) = 15.467, *p* < 0.001, *η*_*G*_^*2*^ = 0.048, with a larger difference between the low-time-pressure and high-time-pressure condition for target-absent (4.56) than for target-present trials (2.14). Search speed was descriptively at the same level for target-absent trials under high time pressure (13.84%/s) and target-present trials under low time pressure (13.41%/s). In this ANOVA, no other effect was significant (*p*s > 0.058).

### Time pressure and target location congruency

**Fig. 3:**
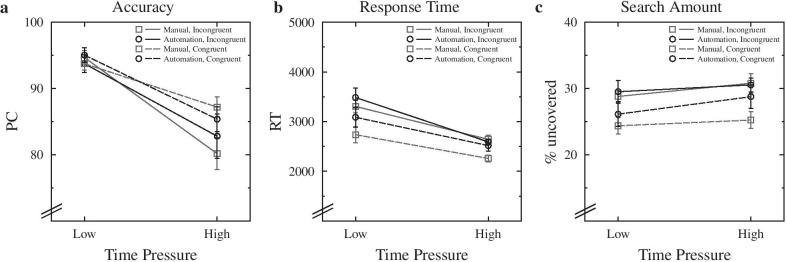
Data for percent correct (**a**), response times (**b**), and search amount (**c**), separately for each time-pressure condition, as a function of automation condition and target location congruency. Note that this analysis was limited to the target-present trials. Error bars represent standard errors of the mean. PC: percent correct, RT: response time

Separating the target-present trials into trials where the target appeared in an expected quadrant of the image or not enabled us to investigate possible effects of target location congruency (target at expected vs. non-expected location) on accuracy, RT, and search amount. We ran a 2 (time pressure: low vs. high, between subjects) × 2 (target location: congruent vs. incongruent, within subjects) × 2 (automation vs. manual, within subjects) mixed ANOVA, and the results are visualized in Fig. 3.

#### Accuracy

For PC, there was again a significant main effect of time pressure, *F*(1,43) = 34.520, *p* < 0.001, *η*_*G*_^*2*^ = 0.283, with less accurate responses under high (83.9%) than under low (94.3%) time pressure. Most interestingly, the main effect of target location congruency was also significant, *F*(1,43) = 6.641, *p* = 0.013, *η*_*G*_^*2*^ = 0.022, with more accurate responses when a target appeared in the predictable location (90.6%) than when it appeared in an incongruent location (88.3%). The interaction of time pressure and location congruency was also significant, *F*(1,43) = 5.707, *p* = 0.021, *η*_*G*_^*2*^ = 0.019, with a congruency effect only present under high (4.8%) but not under low time pressure (0.0%). All other effects were not significant, *p*s > 0.086.

#### Response times

A similar pattern of results emerged for RTs as for PC. That is, the main effect of time pressure was again significant, *F*(1,43) = 22.467, *p* < 0.001, *η*_*G*_^*2*^ = 0.199, with faster responses in the high-time-pressure (2503 ms) than in the low-time-pressure (3154 ms) condition. Interestingly, the main effect of target location congruency was also significant, *F*(1,43) = 22.968, *p* < 0.001, *η*_*G*_^*2*^ = 0.072, with faster responses when the target was in the congruent location (2667 ms) than when it was in the incongruent location (3032 ms). No other effect was significant, *p*s > 0.066.

#### Search amount and search speed

In the ANOVA for search amount, the only significant effect was the effect of target location congruency, *F*(1,43) = 27.396, *p* < 0.001, *η*_*G*_^*2*^ = 0.074, indicating a smaller scanned area when the target appeared in a congruent location (26.1%) than when it appeared in an incongruent location (29.9%). All other effects were not significant, *p*s > 0.156.

For search speed, there was again a significant main effect of time pressure, *F*(1,42) = 8.571, *p* < 0.001, *η*_*G*_^*2*^ = 0.131, with faster search speed under high (13.18%/s) than under low time pressure (15.49%/s). Moreover, there was also a significant main effect of target location congruency, *F*(1,42) = 16.130, *p* < 0.001, *η*_*G*_^*2*^ = . 015, with a faster search speed for target location congruent (14.66%/s) than for incongruent trials (13.92%/s). The interaction of time pressure and target location congruency was also significant, *F*(1,42) = 16.130, *p* < 0.001, *η*_*G*_^*2*^ = . 015, with the target location congruency effect only being present in the low-time-pressure condition (difference: 1.17%/s) but not in the high-time-pressure condition (difference: 0.28%/s).

## Discussion

The present experiment had two main goals. First, we wanted to investigate how time pressure and automation support would affect performance measures and whether visual search amount would change due to these factors in an X-ray luggage-screening task. We generally found performance decrements (for both accuracy and sensitivity) for those participants who were under high time pressure with no evidence for an increased dependence on the automated DSS under high time pressure. Second, we also investigated attention guidance through probability cueing as another potential factor influencing the luggage screening task. Here, we found clear evidence that targets appearing in a target-often location were responded to more accurately, faster, and needed less of a search to find the target. For accuracy, this effect was only present under high time pressure, though, suggesting that time pressure leads to a more heuristic search strategy.

Regarding the general impact of time pressure in this luggage screening visual search task, we will first discuss the effects for target-absent and target-present trials separately. For target-absent trials, time pressure affected RTs, search amount, and consequently also search speed and accuracy. Specifically, participants searched a smaller area of the image in a shorter time, but at an increased search speed. This suggests that participants based their target-absent decisions on less evidence under time pressure compared to situations with more time available. Specifically, it seems like the participants never completed a fully exhaustive search of the images, and this effect was particularly strong under time pressure, despite an increased search speed. This suggests that participants put under time pressure had to terminate their search for targets earlier and, thus, to accept less evidence to base their response on when they made a target-absent decision. The fact that this effect was stronger for target-absent trials than for target-present trials directly supports the predictions derived from the SST model of visual search.

For target-present trials, time pressure increased the search speed but not the search amount. Thus, in this case, it seems that participants tried to scan the same size of search area in a shorter time. This increased the risk of missing targets, though. Note, however, that the results might have looked different if participants had to search for more than one target per bag (i.e., a task with an exhaustive stopping rule), as this would likely have forced them to adapt the search amount under time pressure and to adapt the termination rule of their search. Moreover, the conclusions derived from the search amount and speed results can provide a deeper understanding of the negative impact of time pressure than would have been possible by just having RTs available.

Overall, these findings align well with the findings of McCarley (2009). In this study, effects of different speed–accuracy instructions on performance in inspecting luggage X-rays were studied. In line with our effects of time pressure, an emphasis on speed instead of accuracy led participants not only to inspect less of the image but also to make fewer and shorter fixations, i.e., to increase their search speed. A similar adaptation of search behavior would well explain our findings of a time pressure increased search speed with the mouse-over approach, as well.

Integrating the findings of target-present and absent trials by considering performance measures derived from signal detection theory can help to understand the effects of time pressure in the current experiment even better. Time pressure did not only reduce the sensitivity (d_a_) but also shifted the response criterion (C) to be more conservative. This means that the participants did not just become worse at discriminating between target-present and target-absent images, but also showed more conservative decision making when inspecting the images, i.e., they required more evidence to make a target-present response. Overall, this means that participants became more miss prone under time pressure compared to the condition where they had sufficient time for a careful search.

With respect to practical consequences, this finding seems to be particularly relevant because luggage screening in the real world is a very safety–critical task. That is, one would certainly want to avoid that screeners terminate their search too early, possibly failing to detect some target in an unexpected area—opening the door to some potentially crucial misses, and thus obviously representing a serious risk factor for aviation safety and airport security. In any case, screeners should be sensitized for this issue and instructed and trained to always take the time to perform a fully exhaustive search even if they feel a subjective time pressure, e.g., due to a high passenger volume at their control point.

Providing automation support did not seem to make a big difference with respect to this issue in the present study. Specifically, the present study did not provide evidence that participants were more dependent on the automation under high time pressure, as these two factors did not interact for any of the dependent variables. This contrasts with some earlier studies (Rice et al. 2008, 2010, Rice and Keller 2009; Rice and Trafimow 2012) which showed that with a highly reliable DSS available, time-pressure effects could be ameliorated. Several factors might have contributed to the lack of interaction effects in the present study. That is, earlier studies used a much stronger time-pressure manipulation which just might have forced their participants to depend on their DSS. Moreover, our automation was only moderately reliable (85%) and the task not very difficult, with manual performance exceeding performance with automation support for accuracy. Thus, it seems like not depending on the automation too much actually was sort of functional behavior for our participants. However, besides this lacking effect on overall performance, the response criterion became more liberal with the automation. This makes sense because the automation was mainly false alarm prone. One should consider this effect together with the additive effect of time pressure discussed above which also induced a shift of the response criterion, though in the opposite direction (i.e., more conservative). Consequently, the time-pressure-induced criterion shift also appeared with automation, yet the changed criterion still remained more liberal than when working manually.

Another issue addressed in the present study concerned possible effects of attention guidance through probability cueing and its impact on the visual search and decision making in a luggage screening task. This kind of attentional guidance has been shown to be influenced by past experience (Chun and Jiang 1998), and it seems conceivable that professional screeners also search for targets based on previous experience, i.e., might particularly look at certain areas in a bag where they think or know targets are most prevalent. This kind of target expectancy effect has already been found in more applied areas (e.g., Brockmole and Henderson 2006; Mack and Eckstein 2011; Oliva et al. 2004; Pollatsek et al. 2006; Sha et al. 2018; Shinoda et al. 2001), and the present study extends the list of applied areas where this effect seems to play a role to the context of luggage screening.

More specifically, our results show that detection of critical items was significantly more accurate and faster when the target appeared in the target-often location as compared to other locations. Moreover, as became evident from the search amount data, participants also searched through less space to identify the target before making a target-present decision, suggesting that they made a more effective search in this case. This was also reflected in PC data which suggested that the decision accuracy for targets in the target-often location was less affected by time pressure than the accuracy for targets in other locations. Thus, particularly under high time pressure, participants seemed to have benefitted from the attentional guidance provided by the expectancy manipulation. That is, time pressure might have induced some kind of a proper heuristic search in those cases. Generally, the present findings align well with studies from basic research (e.g., Chun and Jiang 1998; Hoffmann and Kunde 1999; Jiang et al. 2013). Specifically, the spotlight metaphor of visual attention by Miller (1988) seems particularly fitting with the present findings, as in the paradigm used in the present study participants quite literally searched for the target with a spotlight (i.e., the area uncovered by the mouse movements). However, note that in actual real-life luggage screening, target expectancy might be more strongly linked to other items in the bag along which prohibited items are typically hidden. Of course, this kind of expectancy effect is also related to spatial expectancy, albeit an expectancy linked to specific items, and not specific areas as was investigated in the present study. Thus, future studies should investigate whether this kind of target location congruency effect also occurs for this kind of item-specific expectancy.

Finally, another limitation that comes with the nature of our mouse-over approach is that participants had no peripheral information available to them outside the search area during the mouse-over search. Thus, the useful visual field was basically limited to the search area of the mouse, without allowing for peripheral vision to also play a role in the search (see e.g., Wolfe et al. 2017). However, given (a) that object identification is likely one of the most key contributors to task performance in a luggage screening task, and (b) that object identification in the periphery can be quite difficult, we do not think this poses much of an issue to the conclusions drawn based on the current data.

## Conclusion

Overall, the present findings enhance the understanding of detection error sources under time pressure in a naturalistic visual search task. The present research again shows that time pressure should be avoided in safety–critical tasks such as luggage screening. Detection performance deteriorated under high time pressure, while response speed improved, suggesting the presence of a speed–accuracy tradeoff. Moreover, the present study failed to replicate benefits of time pressure on performance in combination with an automated diagnostic aid (e.g., Rice and Keller 2009). Instead, the results confirm other findings (e.g., Wiczorek and Meyer 2019) that providing only moderately reliable automated diagnostic aids are of low value for increasing human–automation performance or can become even countereffective in comparison with human-alone performance if used by humans who master a task very well, anyway. It remains unclear, however, how providing a more accurate DSS would change the present findings. Furthermore, probability cueing of target location seems to influence performance, response time, and search amount in luggage screening through effective attention guidance during the search process.

## Data Availability

Data are available via the Open Science Framework under https://osf.io/9gf86/ Material cannot be made available due to copyright restrictions.

## Appendix

See Tables

**Table 1:** Questionnaire data: NASA-TLX (means and standard deviations)

Variables	Low time pressure		High time pressure	
	Manual	Automation support	Manual	Automation support
Mental demand	12.52 (5.69)	12.88 (6.37)	14.70 (5.95)	15.45 (5.43)
Temporal demand	11.88 (6.20)	12.20 (5.42)	16.75 (3.92)	18.10 (2.45)
Performance	5.12 (4.29)	6.44 (4.58)	8.95 (5.49)	10.90 (5.03)
Effort	12.20 (4.83)	10.64 (6.09)	13.85 (4.11)	15.25 (4.29)
Frustration	6.12 (3.99)	6.76 (5.27)	12.00 (4.35)	12.95 (5.12)

1 and

**Table 2:** Questionnaire data: FMV (means and standard deviations)

Subscale	Low time pressure	High time pressure
Perceived reliability	2.73 (0.57)	2.43 (0.57)
Utility	3.15 (0.63)	2.68 (0.70)
Intention	3.50 (0.43)	3.19 (0.60)
Transparency	2.62 (0.78)	2.63 (0.74)

2.

## Abbreviations

DSS: Decision support system
PC: Percent correct
RT: Response time
